# Supplementary material for: Assessing whether genetic scores explain extra variation in birthweight, when added to clinical and anthropometric measures
Source: BMC Pediatr. 2022 Aug 25;22:504. doi: 10.1186/s12887-022-03554-1 (PMC9414111; doi:10.1186/s12887-022-03554-1)
Supplement: Supplementary file 2 — Additional file 2: Supplementary tables S2-S6. This file contains the supplementary tables for this paper. [file 12887_2022_3554_MOESM2_ESM.docx]

**Supplementary tables**

| **Phenotype** | **Analysed Sample (n=549 trios)** | **Excluded Sample**  **(n=398 trios)** | **p-value** |
| --- | --- | --- | --- |
| Maternal Height (cm) | 165.0 (6.4) | 165.1 (6.2) | 0.776 |
| Maternal Weight (kg) | 76.3 (12.6) | 75.0 (15.1) | 0.169 |
| Gestational Duration (weeks) | 40.1 (1.2) | 40.1 (1.2) | 0.793 |
| Birthweight (g) | 3570 (444) | 3537 (436) | 0.228 |
| Maternal Age (years) | 30.3 (5.3) | 30.6 (5.1) | 0.509 |
| Maternal smoking status (%Yes)* | 14.6 | 10.8 | 0.133 |
| Parity (%1^st^ pregnancy)* | 44.8 | 46.6 | 0.507 |
| Sex of the baby (% Male)* | 52.2 | 51.1 | 0.932 |

**Table S2**: Key characteristics of excluded participants and results of t-tests and chi-square tests for comparison between the analysed sample and the excluded sample.
 * indicates the p-value is for a Chi Square test

| **Outcome Variable** | **Genetic score** | **Change in trait per 1 SD higher genetic score​** | **95% CI** | **Pearson’s Correlation coefficient, r** | **p-value** | **n** |
| --- | --- | --- | --- | --- | --- | --- |
| Offspring birthweight adjusted for sex and gestational age | Maternal genetic score for offspring birthweight (g), with weights adjusted for fetal genotype​ effects | 80.4g | 43.7,117.1 | 0.18 | 2e-05 | 549 |
| Offspring birthweight adjusted for sex and gestational age | Fetal genetic score for birthweight (g), with weights adjusted for maternal genotype effects | 68.0 g | 31.3,104.9 | 0.15 | 0.0003 | 549 |
| Father’s own self-reported birthweight | Paternal genetic score for father’s own birthweight (generated with unadjusted weights) | 59.2g | 27.8, 90.7 | 0.17 | 0.0002 | 504 |

**Table S3**: Associations of maternal, fetal and paternal genetic scores with birthweight

| **Variable** | **Change in birthweight (g) per 1 SD change in X variable** | **95% Confidence Interval** | **t value** | **p-value** |
| --- | --- | --- | --- | --- |
| Intercept | 3727 | 3654, 3778 | 142.9 | <0.001 |
| Maternal age | -44 | -81, -7 | -2.3 | 0.02 |
| Maternal weight | 82 | 41, 122 | 4.0 | <0.001 |
| Mother’s smoking status | -237 | -350, -123 | -4.1 | <0.001 |
| Parity | -219 | -291, -147 | -6.0 | <0.001 |
| Mother’s fasting glucose at 28 weeks’ gestation | 105 | 67, 144 | 5.3 | <0.001 |
| **Mother’s own birthweight** | 103 | 66, 140 | 5.5 | <0.001 |
| **Father’s own birthweight** | 47 | 10, 83 | 2.5 | 0.01 |
| Maternal height | 23 | -16, 62 | 1.2 | 0.25 |
| Paternal height | 60 | 23, 98 | 3.2 | 0.002 |

**Table S4**: Results of a multivariable linear regression model testing the association between birthweight (adjusted for sex and gestational age), maternal clinical characteristics (n=425 parent-offspring trios), and additional parental anthropometric features that capture fetal genetics. R^2^=0.317; Adj-R^2^=0.302

| **Variable** | **Change in birthweight (g) per 1 SD change in X variable** | **95% Confidence Interval** | **t value** | **p-value** |
| --- | --- | --- | --- | --- |
| Intercept | 3870 | 3724, 4016 | 51.9 | <0.001 |
| Maternal age | -43 | -80, -6 | -2.3 | 0.02 |
| Maternal weight | 81 | 41, 122 | 4.0 | <0.001 |
| Mother’s smoking status | -230 | -343, -117 | -4.0 | <0.001 |
| Parity | -226 | -298, -154 | -6.1 | <0.001 |
| Mother’s fasting glucose at 28 weeks’ gestation | 105 | 66, 143 | 5.3 | <0.001 |
| Mother’s own birthweight | 94 | 57, 132 | 4.9 | <0.001 |
| Father ’s own birthweight | 46 | 9, 82 | 2.5 | 0.01 |
| Maternal height | 23 | -16, 61 | 1.1 | 0.25 |
| Paternal height | 60 | 23, 97 | 3.2 | 0.002 |
| **Fetal genetic score for offspring birthweight (adjusted for maternal effects)** | 32 | -4, 67 | 1.8 | 0.080 |

**Table S5**: Results of a multivariable linear regression model testing the association between birthweight (adjusted for sex and gestational age), maternal clinical characteristics (n=425 parent-offspring trios), additional features that capture fetal genetics, and fetal genetic score. R^2^ = 0.329; Adj-R^2^=0.310, (p=0.06 when compared to Model in table S4)

| **Included sample (of those with birthweight and complete phenotype data available)** | **Total N** | **Description of model** | **Change in BW  in g per 1 SD higher X variable (95%CI)** | **R^2^ (95%CI)** |
| --- | --- | --- | --- | --- |
| Max sample with maternal genetic score | 820 | Outcome: birthweight (adjusted for sex and gestational age)  X variable: maternal genetic score | 66 (32-100) | 0.02432  (0.00513, 0.0502) |
| Max sample with fetal genetic score | 646 | Outcome: birthweight (adjusted for sex and gestational age)  X variable: fetal genetic score | 84 (50-117) | 0.03634  (0.0131, 0.0710) |
| Max sample with maternal and fetal genetic scores | 595 | Outcome: birthweight (adjusted for sex and gestational age)  X variable: maternal genetic score | 73 (38-108) | 0.02935  (0.00733, 0.0595) |
| Max sample with maternal and fetal genetic scores | 595 | Outcome: birthweight (adjusted for sex and gestational age)  X variable: fetal genetic score | 78(43-113) | 0.03307  (0.00941, 0.0654) |
| Max sample with maternal, fetal and paternal genetic scores | 549 | Outcome: birthweight (adjusted for sex and gestational age)  X variable: maternal genetic score | 80  (44-117) | 0.036  (0.00966, 0.0697) |
| Max sample with maternal, fetal and paternal genetic scores | 549 | Outcome: birthweight (adjusted for sex and gestational age)  X variable: fetal genetic score | 68  (31-105) | 0.0269  (0.00497, 0.0557) |
|  |  |  |  |  |

**Table S6**: Summary of models describing the contribution of the genetic scores to variation in offspring birthweight in different sample sizes.
